# Viscoelasticity Investigation of Semiconductor NP (CdS and PbS) Controlled Biomimetic Nanoparticle Hydrogels

**DOI:** 10.3389/fchem.2021.816944

**Published:** 2022-01-19

**Authors:** Dan Zhao, Wang Zhang, Zhi-Zhou Chen

**Affiliations:** ^1^ School of Marine Sciences, Ningbo University, Ningbo, China; ^2^ College of Electrical and Electronic Engineering, Wenzhou University, Wenzhou, China

**Keywords:** viscoelasticity, colloidal nanoparticles, hydrogels, self-assembly, macroscale mechanics

## Abstract

The viscoelastic properties of colloidal nanoparticles (NPs) make opportunities to construct novel compounds in many different fields. The interparticle forces of inorganic particles on colloidal NPs are important for forming a mechanically stable particulate network especially the NP-based soft matter in the self-assembly process. Here, by capping with the same surface ligand L-glutathione (GSH), two semiconductor NP (CdS and PbS) controlled biomimetic nanoparticle hydrogels were obtained, namely, CdS@GSH and PbS@GSH. The dependence of viscoelasticity of colloidal suspensions on NP sizes, concentrations, and pH value has been investigated. The results show that viscoelastic properties of CdS@GSH are stronger than those of PbS@GSH because of stronger surface bonding ability of inorganic particles and GSH. The hydrogels formed by the smaller NPs demonstrate the higher stiffness due to the drastic change of GSH configurations. Unlike the CdS@GSH hydrogel system, the changes of NP concentrations and pH value had great influence on the PbS@GSH hydrogel system. The higher the proportion of water in the small particle size PbS@GSH hydrogel system, the greater the mechanical properties. The stronger the alkalinity in the large particle size PbS@GSH hydrogel system, the greater the hardness and storage modulus. Solution˗state nuclear magnetic resonance (NMR) indicated that the ligand GSH forms surface layers with different thickness varying from different coordination modes which are induced by different semiconductor NPs. Moreover, increasing the pH value of the PbS@GSH hydrogel system will dissociate the surface GSH molecules to form Pb^2+^ and GSH complexes which could enhance the viscoelastic properties.

## Introduction

Because of the charming performance exhibited by materials as they progress from the atomic to the molecular scale and approach extended solids, soft matter fabricated from various metal and semiconductor NPs in colloidal suspensions has gained considerable attention in recent decades (Boles et al., 2016; Li et al., 2021; Mao et al., 2016; Scalise et al., 2018; Al˗Johani et al., 2017; Algar et al., 2019; Love et al., 2015; Gu et al., 2021). Colloidal nanoparticles (NPs), a kind of soft matter, formed by water-soluble inorganic nanoparticles, have been demonstrated to afford an opportunity to merge the nanoscale world with macroscopic-sized materials that can be readily processed and operated while retaining nanoscale performance (Kotov and Weiss, 2014; Bhattacharya and Samanta, 2016; Yip et al., 2019). Despite this, the relationship between the NP structure and the “size-tunable” characters has not been thoroughly studied. These small crystals are materials with high surface area, and their surface plays a key role in the material’s physical and chemical behavior. Therefore, understanding and characterizing the surface of semiconductor NPs is necessary to reasonably control and adjust their properties (Han et al., 2015; Yang et al., 2017).

Colloidal NPs generally consist of three regions: an inorganic metal core, an NP surface layer, and organic ligands, which are covalently bonded to the NP surface (Kotov, 2010). Influences from NP surfaces and cores are actually not separate from each other, whether synergistic or counteracting in terms of physical features. The interaction between inorganic metal NP cores and surfaces is central to a broad spectrum of biological, chemical, and physical phenomena (Shavel et al., 2006; Min et al., 2008; Hens and Martins, 2013; Batista-Silvera et al., 2015; Zhou et al., 2016; Rechberger and Niederberger, 2017; Chu et al., 2018; Piveteau et al., 2018). Insight into these interactions is very important for technology realization of nanoscale synthesis and engineering the supermolecular structure of self-assembly NPs with different dimensions, collective characteristics at the nanoscale, and predictive biological responses to NPs. The viscoelasticity of gels composed of NPs with short organic ligands exhibited significant differences from that of other gels, as their mechanical behavior involves the interaction of both the surface ligands and the NP core while preserving their reconfigurability, and the relevant research and a universal model for the surface coordination of colloidal NPs were given by Zhou’s group (Zhou et al., 2018; Zhang et al., 2019). But the factors affecting the mechanical properties of gel have not been systematically studied and discussed so far.

In this work, we synthesized a series of NP hydrogels from different semiconductor NPs (CdS and PbS) capped with the same surface peptide layer of L-glutathione (GSH), namely, CdS@GSH and PbS@GSH, to investigate the influence of different inorganic metal nuclei on the conformation of surface ligands and their effect on the mechanical behavior of their corresponding hydrogels. The effects of size, concentration, and pH value of NPs on the viscoelastic properties of colloidal suspensions were investigated. The results show that viscoelastic properties of CdS@GSH are stronger than those of PbS@GSH because of stronger surface bonding ability of inorganic particles and GSH. The hydrogels consisting of the smaller NPs show the highest stiffness because of the significant changes in GSH configurations. Unlike the CdS@GSH hydrogel system, the changes of NP concentrations and pH value had great influence on the PbS@GSH hydrogel system. The higher the proportion of water in the small particle size PbS@GSH hydrogel system, the greater the mechanical properties. The stronger the alkalinity in the large particle size PbS@GSH hydrogel system, the greater the hardness and storage modulus. Solution˗state nuclear magnetic resonance (NMR) indicated that the ligand GSH forms surface layers with different coordination modes depending on different semiconductor NPs. In the CdS@GSH hydrogel system, the surface ligand GSH using the functional group (–SH, –COOH, and –NH_2_) coordinated with Cd^2+^ to form a strong three-point bonding mode to strengthen the mechanical properties, which was consistent with the universal model reported by Zhou’s group. But for the PbS@GSH hydrogel system, with the surface ligand GSH, the ^13^C chemical splitting of C_β_ and C_β_΄ carbon represents the presence of (GSH)S-Pb and (GSH)_2_S_2_-Pb complexes on the surface of Pb@GSH NPs with different coordination modes. The existence of these two different coordination species weakens the mechanical properties of Pb@GSH hydrogels. Moreover, increasing the pH value will dissociate the surface species of the PbS@GSH hydrogel system to form Pb^2+^ and GSH complexes which could enhance the viscoelastic properties.

## Results and Discussion

### Preparation and Characterization of GSH-Stabilized CdS and PbS NPs and Their Corresponding Hydrogels

**FIGURE 1:**
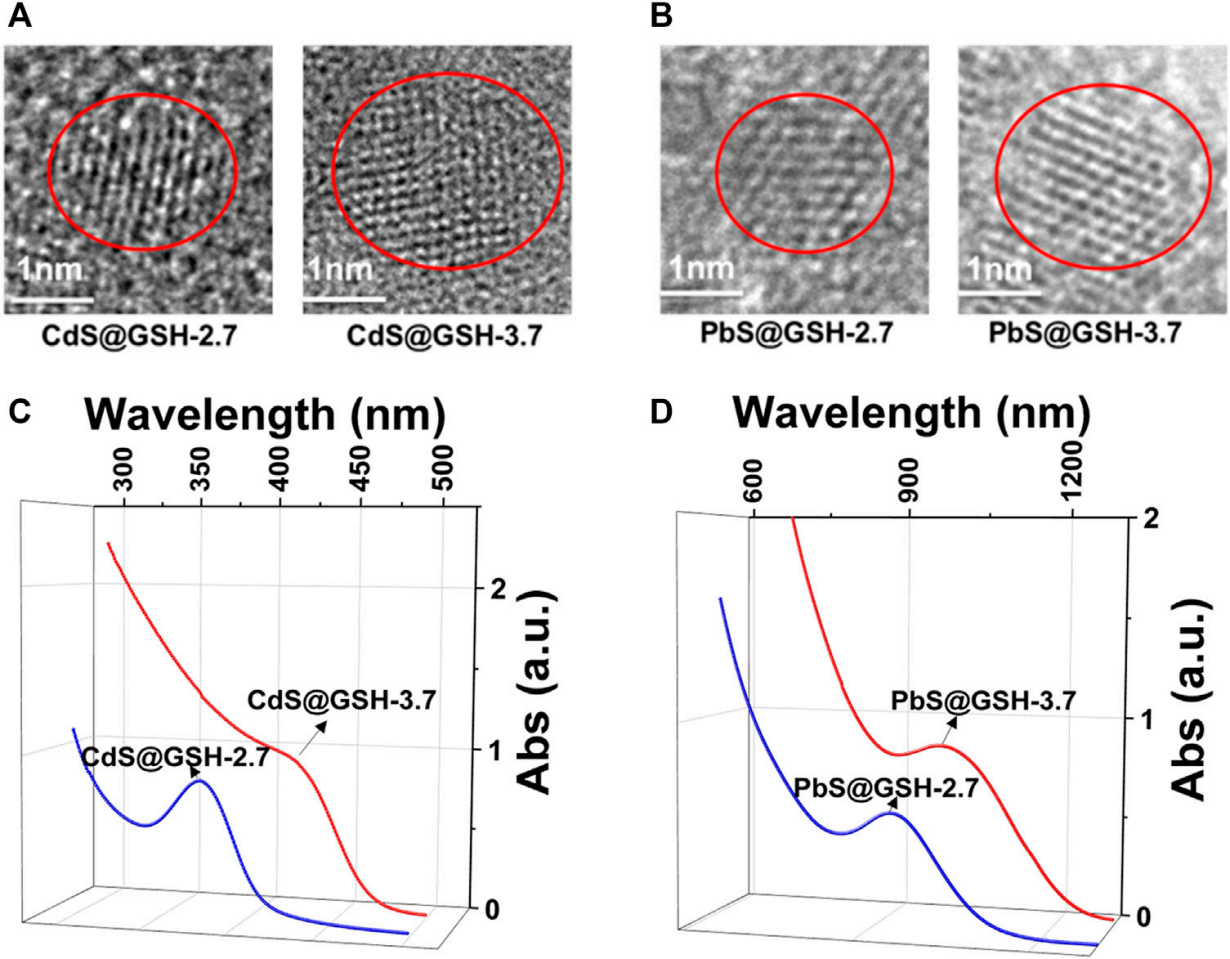
HRTEM images of 2.7 and 3.7 nm NPs: **(A)** CdS@GSH and **(B)** PbS@GSH. Absorption spectra of NP dispersions: **(C)** CdS@GSH and **(D)** PbS@GSH.

Here, CdS and PbS NPs, modified by organic peptide L-glutamyl-L-cysteinyl-glycine (GSH), were synthesized in aqueous solution. Owing to the ability to form multiple intermolecular hydrogen bonds, GSH was selected as an NP surface ligand. NPs with different inorganic core diameters (2.7 and 3.7 nm) were made by regulating the refluxing temperature and time. (After refluxing for 3 h under 105°C, CdS@GSH NPs of 2.7 nm were obtained, and CdS@GSH NPs of 3.7 nm were obtained after refluxing for 10 h under 105°C; PbS@GSH NPs of 2.7 nm were obtained after reflux for 1.5 h under 50°C, and PbS@GSH NPs of 3.7 nm were obtained after refluxing for 7.5 h under 50°C.) Aqueous dispersions of CdS and PbS NPs with sizes about 2.7 and 3.7 nm could remain stable for 24 months under −4°C. High-resolution transmission electron microscopy (HRTEM) was applied to estimate the nanocrystal size and monodispersity of NPs. HRTEM images give a visual representation of GSH-stabilized CdS and PbS NPs with the average size of 2.7 and 3.7 nm (Figures 1A,B, Supplementary Figures S1, S2). At sufficiently low concentrations of CdS and PbS, the stabilizer allows for the crystallization of narrow-sized NPs featuring sharp absorption and emission peaks. After maturity, GSH-modified CdS and PbS NPs with a maximum of absorption at 377 nm (small size) and 410 nm (large size) for CdS and 908 nm (small size) and 990 nm (large size) for PbS (Figures 1C,D) were characteristic of the first excitonic state. Based on the correlations between the location of the first absorption feature and the diameter of NPs, 377 nm (small size) and 410 nm (large size) for CdS and 908 nm (small size) and 990 nm (large size) for PbS correspond to NPs with a diameter of 2.7 and 3.7 nm the same as in HRTEM images (Sapra and Sarma, 2004; Cheng and Li, 2017). The PXRD pattern of CdS@GSH and CdS@GSH NPs at different sizes and pH values imply the pure phases of NPs (Supplementary Figure S3).

**FIGURE 2:**
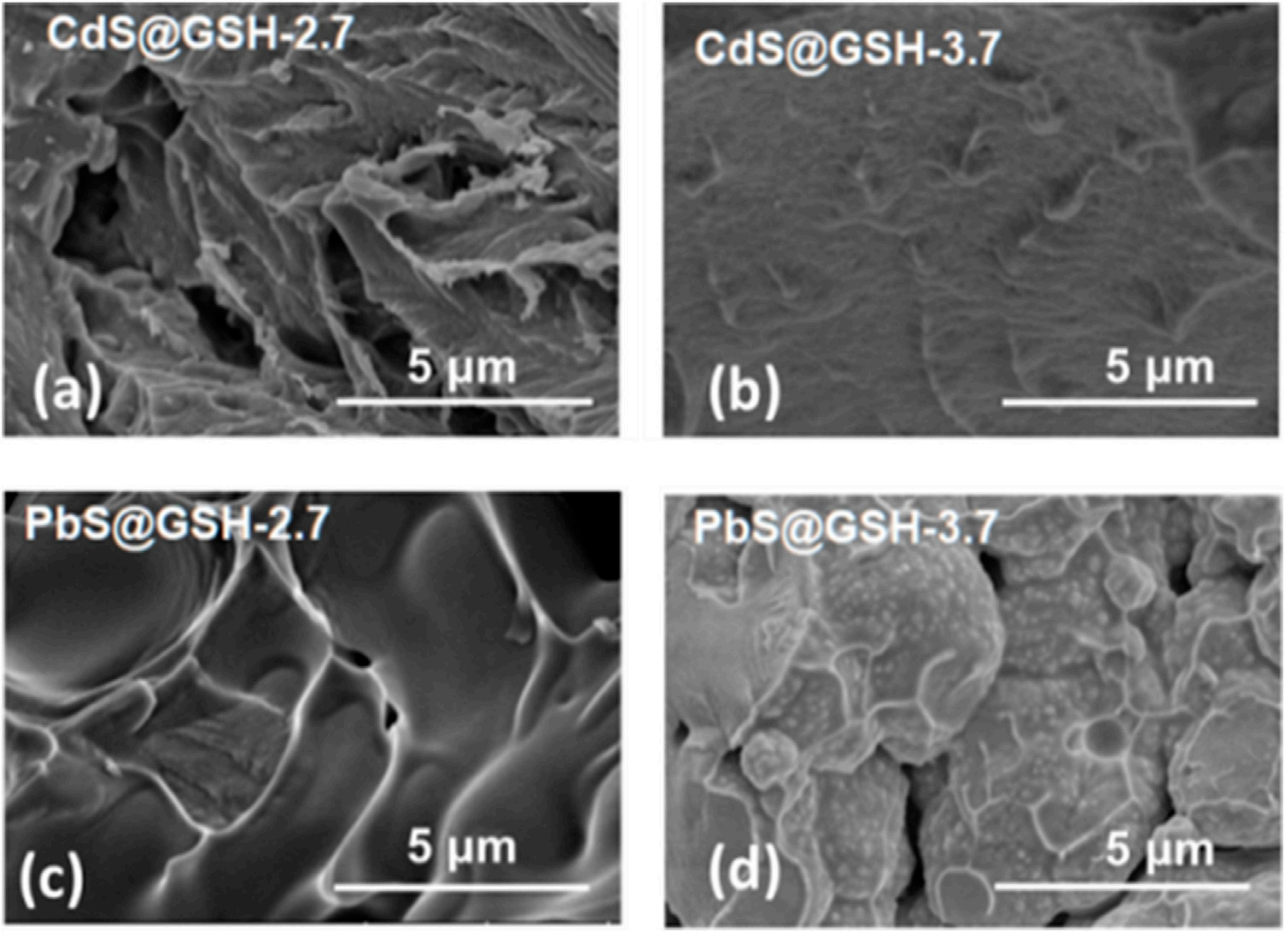
SEM images of CdS@GSH **(A, B)** and PbS@GSH **(C, D)** hydrogels.

During the synthesis process, lots of ligands and precursor compounds that formed a dynamic equilibrium for the concentration of each species by adsorption and desorption with NPs are introduced into the nanocrystal. These unbound compounds, created by the non-covalent forces among the NPs, maintain the stability of NP superficial configuration, finally enriching their chemical and physical characteristics. To remove them from the colloidal solution, one volume of isopropanol was added to one volume of NP aqueous dispersion to prevent the chemical damage of surface ligands and etch the NP surface. After addition of isopropanol, the nanocrystal solution was separated by centrifugation at 3,000 r.p.m./min for 3 min and drying under vacuum conditions. By adding 24 w/w% deionized water into the dried NP solid, a voluminous, intensely luminescent gel with high quantum yields of about 31% was fabricated. Scanning electron microscopy (SEM) images show slight differences. For CdS@GSH hydrogels, the morphology of the smaller size sample is more of a brittle solid than the NP gel in the larger sample (Figures 2A,B). But for PbS@GSH hydrogels, the morphology of the larger size sample is more of a brittle solid than the NP gel in the smaller sample (Figures 2C,D).

### Viscoelastic Properties of Different NP Size–Controlled CdS@GSH and PbS@GSH Hydrogels

**FIGURE 3:**
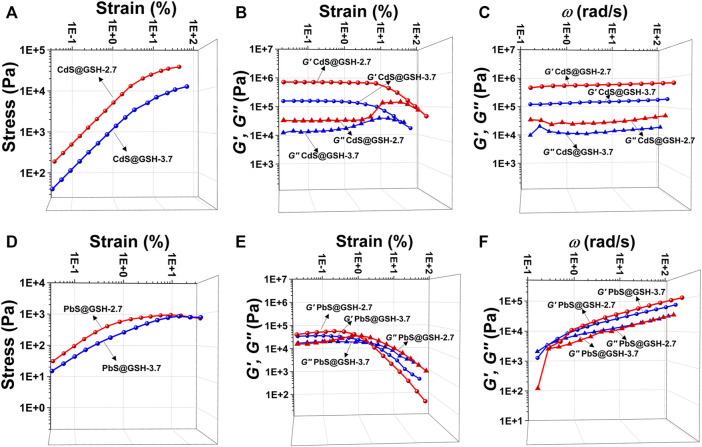
Mechanical characterization of different hydrogels. Measurement of oscillatory stress/strain of CdS@GSH hydrogels **(A)** and PbS@GSH hydrogels **(D)**. Measurement of continuous step moduli/strain of CdS@GSH hydrogels **(B)** and PbS@GSH hydrogels **(E)**. Each measurement was carried out three times with the same sample. Rheological dynamic oscillatory frequency sweep tests of CdS@GSH hydrogels **(C)** and PbS@GSH hydrogels **(F)**. The shear dynamics for each hydrogel was measured from low to high strain starting at 0.01–25% at a frequency of 6.28 rad/s. The rheological dynamic oscillatory frequency sweep measurements were performed with a parallel fixed plate (diameter 25 mm) at a strain value of 0.01%.

When the NPs form into gels, the interactions between inorganic metal NP cores and surfaces as well as the size of NPs are central to their collective forces in terms of their viscoelasticity. Oscillatory stress/strain tests on freshly prepared gels of CdS@GSH and PbS@GSH NPs of different sizes demonstrated that the stress linearly increased with the amplitude of the strain applied to the sample during the initial phase. As for CdS@GSH hydrogels (Figure 3A), the gel made from the smaller NPs (size = 2.7 nm) exhibited higher stiffness and shear modulus for all strains than the larger one (3.7 nm) which were associated with the combination of strong attraction interactions between the NPs. However, different from CdS@GSH hydrogels, poor stiffness and energy dissipation were observed for PbS@GSH NP hydrogels, and particle size dependence of viscoelastic properties was weaker (Figure 3D). At high strains, a clear non-linear behavior is observed as the gel starts to flow. The critical strain where upon the gel starts to flow can be identified by the flex point in the strain dependences for the storage modulus *G*′ and the loss modulus *G*″. This flow point is at about 9% strain for CdS@GSH hydrogels, while PbS@GSH NP hydrogels begin to flow at about 1% strain (Figure 3B). Moreover, the storage modulus values of the PbS@GSH NP gels decreased more rapidly, meaning that once the structure of the gel was disrupted, the interaction between the PbS@GSH NPs was weaker than that between the CdS@GSH NPs (Figure 3E). The variation of dynamic mechanical properties with cyclic frequency *ω* could show viscous deformations in these fluids. CdS@GSH hydrogels indicated a plateau for both moduli and over the entire range of frequencies *G′* > *G″* (Figure 3C). And both moduli increase with *ω*. But for PbS@GSH NP hydrogels, there was a little difference between *G′* and *G″*, and values of stiffness, shear modulus, storage moduli, and loss moduli are lower (Figure 3F).

### Influence of Concentration and pH Value on Macroscale Mechanics of CdS@GSH and PbS@GSH Hydrogels

The mass ratio of DI water and the pH value of nanocrystal solution were turned to further explore the unique viscoelastic properties of CdS@GSH and PbS@GSH hydrogels. For the CdS@GSH-2.7 hydrogel system, adjusting the mass ratio of DI water could not affect the morphology and the mechanical properties of gels (Supplementary Figures S4A, S5A–C). For the CdS@GSH-3.7 hydrogel system, increasing the mass ratio of DI water could reduce the hardness and stress of gels, which may not be significant according to the little change in the shear modulus, storage moduli, and loss moduli (Supplementary Figures S4B, S5D–F). In addition, increasing the pH value could reduce the mechanical properties of CdS@GSH-2.7 hydrogel systems lightly (Supplementary Figures S6A–C) but improve the mechanical properties of large-size hydrogel systems (Supplementary Figures S6D–F).

**FIGURE 4:**
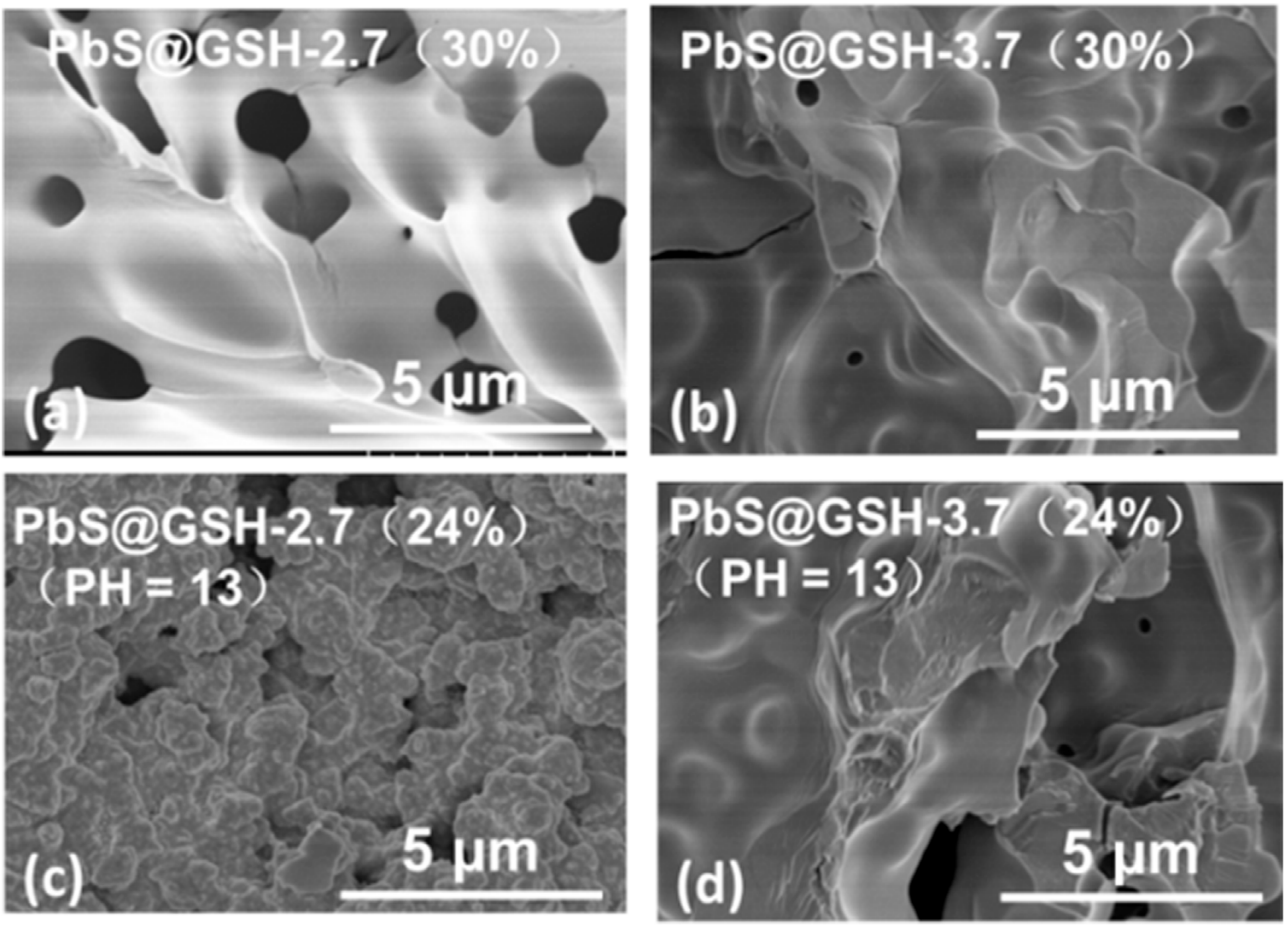
SEM images of PbS@GSH hydrogels under different concentrations **(A,B)** and pH values **(C,D)**.

**FIGURE 5:**
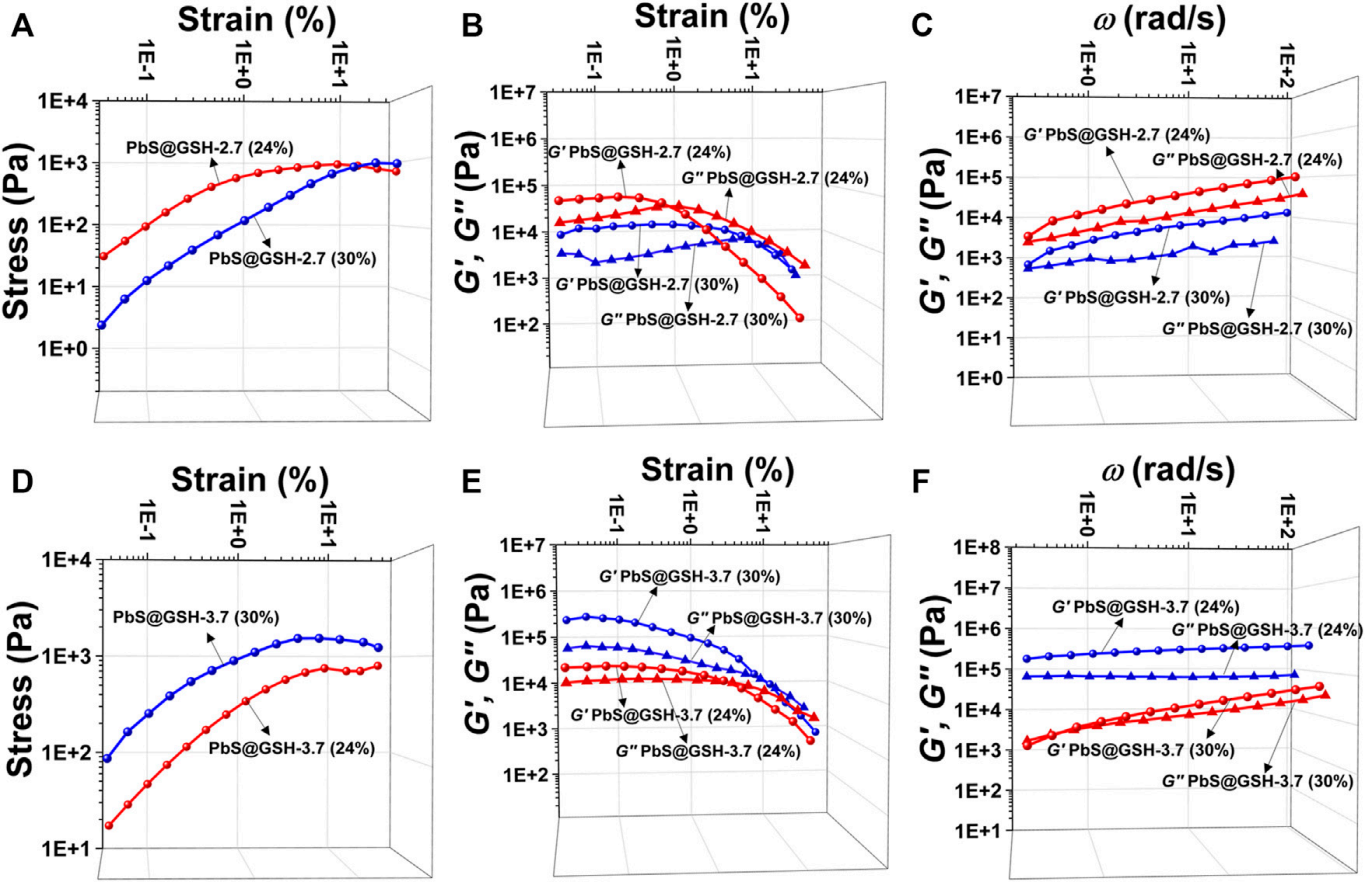
Tests of mechanical properties of different sizes of PbS@GSH hydrogels at different concentrations. Measurements of stress/strain **(A, D)**, continuous step moduli/strain **(B, E)**, and rheological dynamic oscillatory frequency sweep tests **(C, F)**. Each measurement was performed three times with the same sample. The shear dynamics for each hydrogel was measured from low to high strain starting at 0.01–25% at a frequency of 6.28 rad/s. The rheological dynamic oscillatory frequency sweep measurements were performed with a parallel fixed plate (diameter 25 mm) at a strain value of 0.01%.

For the PbS@GSH hydrogel system, increasing the mass ratio of DI water and pH value has a significant impact on the morphology and mechanical performance of the system. SEM images show that the morphology of the smaller size sample under lower concentration is more of a brittle solid than the NP gels from the larger sample (Figures 4A,B). Moreover, for the small particle size PbS@GSH hydrogel system, when the mass ratio of DI water was turned to 24 w/w%, the gel displayed higher stiffness and shear modulus when the flow point is at ∼ 5% (Figure 5A). But for the large particle size PbS@GSH-3.7 hydrogel system, when the mass ratio of DI water was turned to 30 w/w%, the gel showed the higher stiffness and shear modulus for all strains (Figure 5D). Apparently, non-linear behavior when the gels began to flow showed that the storage modulus values of 24 w/w% gel decreased faster than those of the 30 w/w% gel independently of NP size. So, the interaction between the 30 w/w% gels is stronger than that between the 24 w/w% gels when the structure of the gels is disrupted. The variation of dynamic mechanical properties of the smaller NPs is remarkably different from those of the larger NPs when the mass ratio of DI water is changed. For the smaller NPs, 24 w/w% gel shows higher values of stiffness, shear modulus, storage moduli, and loss moduli (Figures 5B,C). However, the changing tendency is completely opposite of that of the larger NPs (Figures 5E,F). All gels were demonstrated over the entire range of frequencies *G′* > *G″*.

**FIGURE 6:**
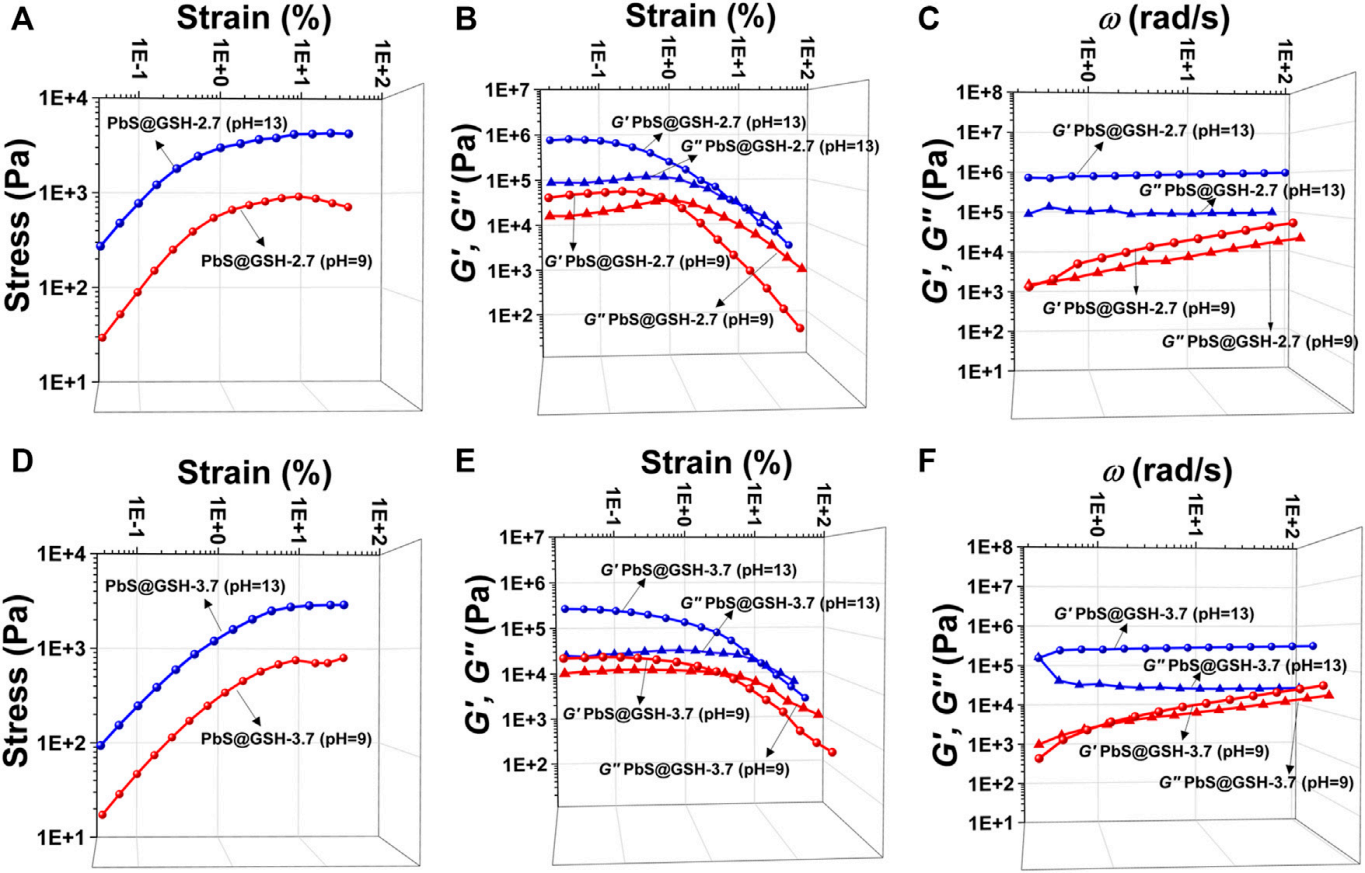
Tests of mechanical properties of different sizes of PbS@GSH hydrogels at different pH values. Measurements of stress/strain **(A, D)**, continuous step moduli/strain **(B, E)**, and rheological dynamic oscillatory frequency sweep tests **(C, F)**. Each measurement was performed three times with the same sample. The shear dynamics for each hydrogel was measured from low to high strain starting at 0.01–25% at a frequency of 6.28 rad/s. The rheological dynamic oscillatory frequency sweep measurements were performed with a parallel fixed plate (diameter 25 mm) at a strain value of 0.01%.

The pH value of PbS@GSH NP solution was by adding NaOH (2 mM) after the reaction. Obviously, increasing the alkalinity of gel system could strengthen the stiffness and shear modulus for all strains no matter the NP size. SEM images show that the morphology of the samples under higher pH value (pH = 9) is more like a brittle solid compared to the NP gels under pH = 9 which is independent of particle size (Figures 4C,D). At high strains, this flow point is at about 0.5% strain for a lower pH PbS@GSH hydrogel system, while the flow point is at about 1.2% strain for a higher pH PbS@GSH hydrogel system (Figures 6A,D). Additionally, the storage modulus values of the low pH hydrogel systems decreased more rapidly than those of the higher pH hydrogel system (Figures 6B,E). The result indicated that once the structure of the gel is destroyed, the interactions between the PbS@GSH NPs at the lower pH value are weaker than those at the higher pH value. Besides, viscous deformations were acquired from the variation of dynamic mechanical properties with *ω* (Figures 6C,F). High pH NPs revealed a plateau for both moduli and over the entire frequency range *G′* > *G″*, and the magnitude of *G′* exceeds 10^6^ Pa. For lower pH NPs, both moduli increase with *ω*. Lower pH NPs again showed lower values of stiffness, shear modulus, storage moduli, and loss moduli compared to higher pH NPs.

### Effect of pH Value on the Molecular Configuration of the Surface GSH Ligand

Surface ligands enable control of colloidal stability and self-assembly of NPs. Considering the pH value has great influence on the mechanical properties of the gels, the nuclear magnetic resonance (NMR) techniques have been used to investigate the molecular configuration of the GSH ligand on the surface of Cd@GSH and PbS@GSH NPs under different pH values. Here, we chose the smaller particle size (2.7 nm) hydrogel system as the research object because the change trend of gel properties is independent of particle size.

**FIGURE 7:**
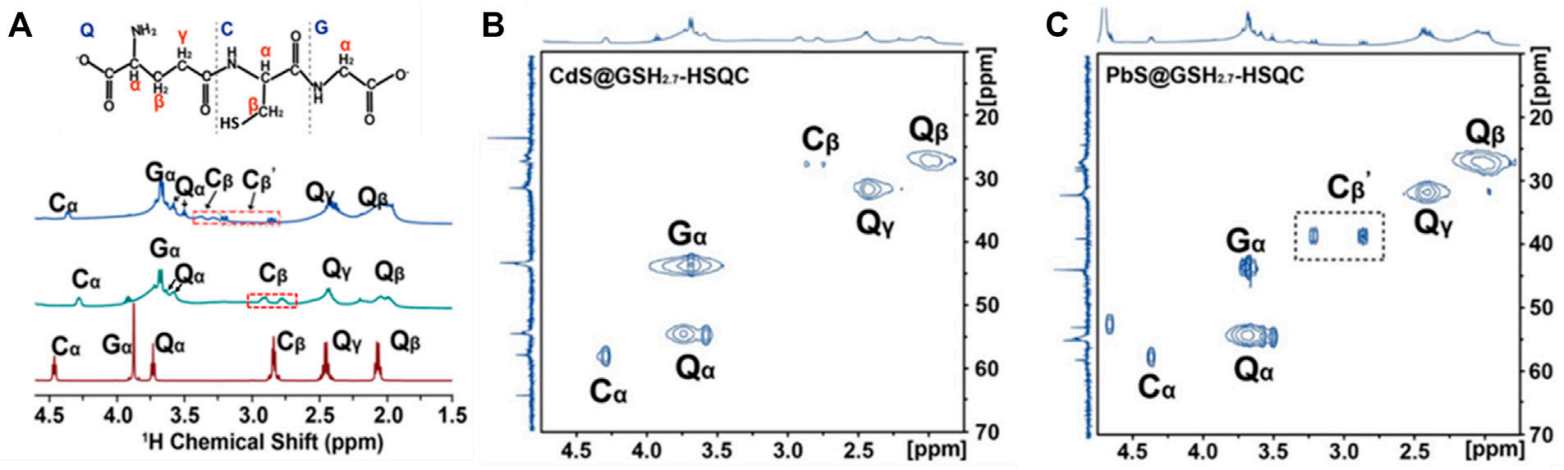
**(A)** Molecular structure of the GSH ligand at the NP surface. Amino acids in GSH are represented by a single-letter code: glutamyl, Q; glycinyl, G; cysteinyl, C. **(A)** Solution-state NMR spectra of PbS@GSH hydrogels (blue line), CdS@GSH hydrogels (green line), and GSH hydrogels (red line). ^1^H–^13^C HSQC spectra of **(B)** 2.7 nm CdS@GSH and **(C)** 2.7 nm PbS@GSH.

From the ^1^H NMR spectroscopy of GSH and hydrogels, the C_α_ signals between 4.3 and 4.4 ppm, Q_α_ signals between 3.5 and 3.7 ppm, and C_β_ signals between 2.7 and 3.5 ppm standardize three-point bonding, indicating that the functional group (–SH, –COOH, and –NH_2_) of GSH coordinated with Cd^2+^ or Pb^2+^ ions by coordination bonds, respectively (Figure 7A). Based on the elemental analysis results and the NMR result, the mass ratios of bound ligands and the inorganic core can be obtained and provided in Supplementary Table S1. In addition, the IR spectra of free ligand GSH and PbS@GSH and CdS@GSH NPs show that –SH and –COOH with a single peak appear at 2,525 and 1710 cm^−1^, and −NH_2_ shows an equal intensity doublet at about 3,300 cm^−1^; these signals disappeared when GSH bound to the surface of the nanoparticles (Supplementary Figure S7). Subsequently, ^1^H-^13^C heteronuclear single quantum coherence (HSQC) was applied to differentiate the species of surface GSH ligand.

The identification of ligand types using HSQC experiments works well for a system with multiple components. In the Cd@GSH system, the ^13^C chemical shift of Q_α_ carbon is ∼51 ppm, and that of Q_β_ methylene carbon is ∼44 ppm. These resonances shifted downfield slightly when GSH bound to the NPs. This means that the Cd@GSH solution contains both (GSH)COO-Cd complexes and (GSH)N-Cd complexes as Cd binds to the carboxylate oxygen and the amino group of GSH which is consistent with the previous report (Zhou et al., 2018) (Figure 7B, Supplementary Figures S8A,B). Besides, the ^13^C chemical splitting of C_β_ carbon means there exist (GSH)S-Cd complexes on the surface of NPs. Different from the Cd@GSH system, the ^13^C chemical splitting of C_β_ and C_β_΄ carbon in the Pb@GSH system represents the presence of (GSH)S-Pb and (GSH)_2_S_2_-Pb complexes on the surface of Pb@GSH NPs with different coordination modes (Figure 7C and Supplementary Figure S8C).Therefore, we further focus on the microstructure of PbS@GSH and different pH value effects on macroscopic mechanical properties.

**FIGURE 8:**
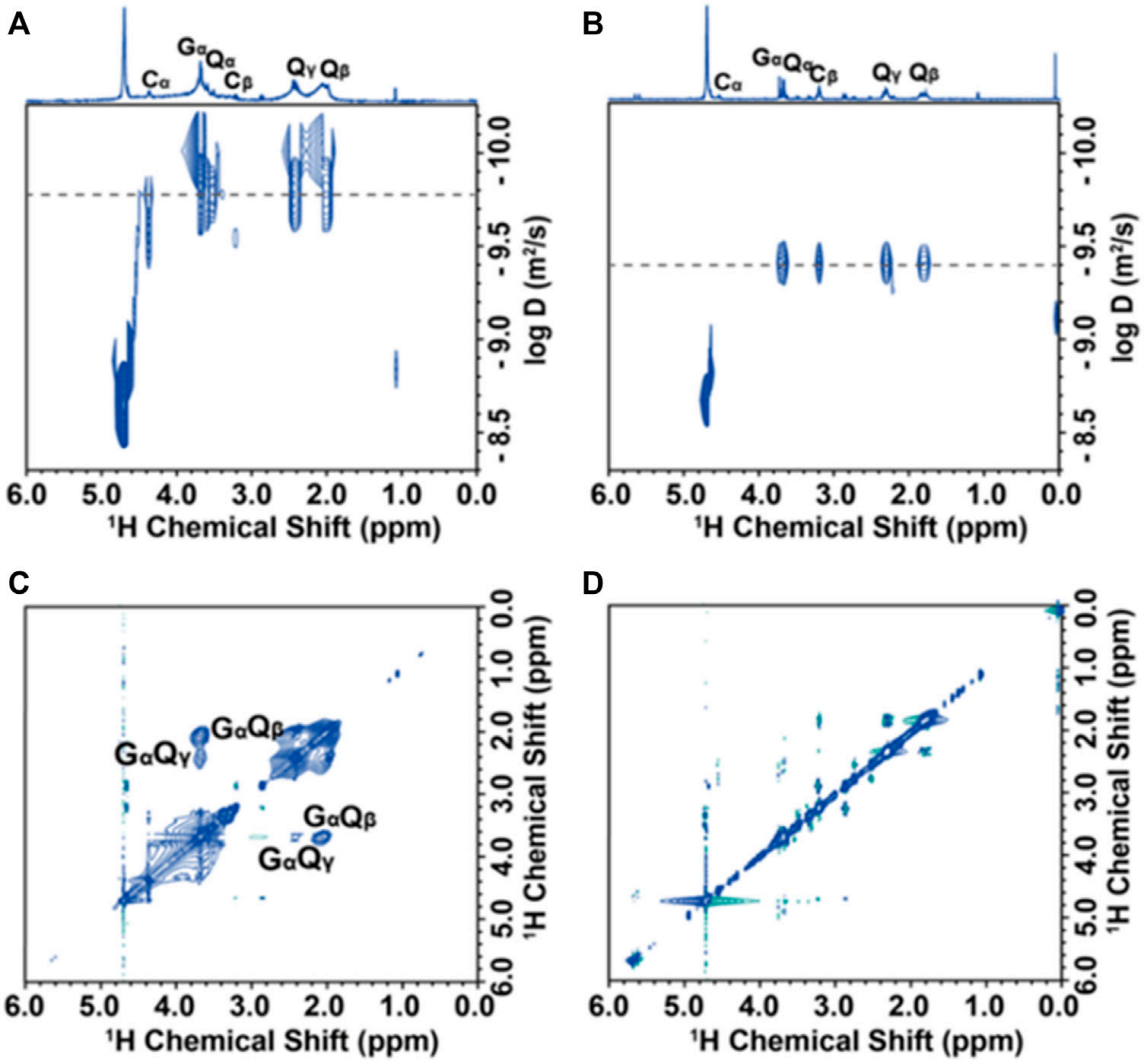
Solution-state NMR analysis of PbS@GSH NP dispersions: DOSY spectra **(A, B)** and ^1^H–^1^H NOESY spectra **(C, D)** of PbS@GSH NPs of 2.7 nm at pH = 9 and pH = 13, respectively.

DOSY (diffusion-ordered NMR spectroscopy) can distinguish species on the basis of their diffusivity and uncover interactions and dispersion in suspensions. Generally, the diffusion coefficient of the ligand bound to the NP surface will be about an order of magnitude smaller than that of the free molecule. In PbS@GSH dispersions, residual water has the same diffusion coefficient as 1.7 × 10^−9^ m^2^s^−1^ at pH = 9, which is in line with the value reported by the literature (Zhang et al., 2019) (Figure 8A), and the GSH ligand does have a small diffusion coefficient (1.7 × 10^−10^ m^2^s^−1^), which can be used to calculate its hydrodynamic diameter according to the Stokes–Einstein equation (Fritzinger et al., 2010). The hydrodynamic diameter of the NP solution is 2.9 nm, which is equal to the sum of the hard core diameter and the thickness of the outer ligand of the NP, indicating that the ligand can effectively coat on the NP surface. Unlike NP dispersions (pH = 9), the ^1^H NMR spectrum resonance of NP dispersions (pH = 13) is obviously narrow and sharp, and the diffusion coefficient (3.98 × 10^−10^ m^2^s^−1^) is also significantly increased, showing free composition is dominant (Figure 8B).

NOESY (nuclear Overhauser effect spectroscopy) was further used to demonstrate the interaction of ligands in NP solution. The binding ligands and the hard cores of NPs shifted together at slow tumbling rates like macromolecules, indicating that their ^1^H signals produce negative NOE cross-peaks with the same sign as their diagonal peaks (Figure 8C, blue contour). The cross-peaks of G_α_Q_β_ and G_α_Q_γ_ PbS@GSH systems at pH = 9 confirm that the GSH ligand does have a strong effect on the NP surface. However, there is no obvious cross-peak in NP dispersions when the pH adjusted to 13, indicating the ligand interacts weakly with the NP and moves freely (Figure 8D).

## Conclusion

In summary, a series of NP hydrogels induced by different semiconductor NPs (CdS and PbS) capped with the same surface peptide layer of glutathione (GSH) were obtained to investigate the mechanical behavior of their corresponding gels. The study shows that viscoelastic properties of CdS@GSH are stronger than those of PbS@GSH because of stronger surface bonding ability of inorganic particles and GSH. In addition, we systematically discussed the influence of nanoparticle sizes, concentrations, and pH value on the viscoelastic properties of colloidal suspensions. The hydrogels formed by the smallest NPs show the highest stiffness, which was attributed to the drastic change of GSH configurations. Unlike CdS@GSH hydrogel systems, the changes of NP concentrations and pH value had great influence on the PbS@GSH hydrogel system. The higher the proportion of water in the small particle size PbS@GSH hydrogel system, the greater the mechanical properties. The stronger the alkalinity in the large particle size PbS@GSH hydrogel system, the greater the hardness and storage modulus. Besides, the NMR uncovered that the ligand GSH forms surface layers with different coordination modes depending on different semiconductor NPs. In the CdS@GSH hydrogel system, the surface ligand GSH using the functional group (–SH, –COOH, and –NH_2_) coordinated with Cd^2+^ to form strongly three point bonding mode to strengthen the mechanical properties, which is consistent with the literature reported in Nature Communications by Zhou’s group. But for the PbS@GSH hydrogel system, the ^13^C chemical splitting of C_β_ and C_β_΄ carbon represents the presence of (GSH)S-Pb and (GSH)_2_S_2_-Pb complexes on the surface of Pb@GSH NPs with different coordination modes. The existence of these two different coordination species weakens the mechanical properties of Pb@GSH hydrogels. Moreover, increasing the pH value will dissociate the surface species of the PbS@GSH hydrogel system to form Pb^2+^ and GSH complexes which could enhance the viscoelastic properties. To our knowledge, this is the first example for systematic study of the viscoelastic properties of PbS@GSH hydrogels.

## Experimental Section

### General Information

All chemicals and solvents obtained from commercial sources without further purification are of reagent grade. L-Glutathione (GSH) was purchased from J&K. Aluminum telluride (Al_2_Te_3_) powder was purchased from KYD materials. Lead(II) perchlorate trihydrate (Pb(ClO_4_)_2_·6H_2_O) and thioacetamide were from Alfa-Aesar. Cadmium perchlorate hexahydrate (Cd(ClO_4_)_2_·6H_2_O) was obtained from Macklin. Isopropanol was obtained from Sigma-Aldrich. These chemicals were used as received. Ultraviolet and visible spectroscopy (UV–Vis) absorption spectra were measured at room temperature on the PerkinElmer Lambda 25 UV–Vis spectrophotometer. SEM images were tested on a Hitachi SU˗8010 scanning electron microscope, and HRTEM images were taken on an FEI˗T20F transmission electron microscope at 200 kV. Fluorescence (PL) spectra were obtained on Horiba FluoroMax 4, and in the measurements of emission and excitation spectra, the pass width is 10 nm. Elemental analyses (EA) for C, H, and N were performed on a PerkinElmer 240C elemental analyzer at the analysis center of Nanjing University. FT-IR spectra were recorded using KBr discs in the range of 400–4000 cm^−1^ on a Bruker Vector 22 FT-IR spectrophotometer. Thermogravimetric analyses (TGAs) were carried out on a Mettler Toledo (TGA/DSC1) thermal analyzer under nitrogen at a heating rate of 10°C min^−1^. Powder X-ray diffraction (PXRD) data were collected on a Bruker D8 Advance X-ray diffractometer with Cu Kα (*λ* = 1.5418 Å) radiation. All the measurements were carried out under the same experimental conditions.

### Preparation of L-GSH–Stabilized Nanoparticles (CdS@GSH and PbS@GSH)

CdS@GSH. Briefly, 0.8 g (0.019 M) of Cd(ClO_4_)_2_·6H_2_O and 1 g (0.03125 M) of L-GSH were dissolved in 100 ml deionized water, and then the pH of the solution was adjusted to 9.0 by adding 2 M NaOH. The solution was placed into a three-necked flask and bubbled with nitrogen about 30 min, followed by the fast addition of aqueous thioacetamide solution (1.5 ml, 10 mM). CdS@GSH of different sizes can be obtained by controlling the heating time at 105°C. And the solution turned from colorless to yellow. CdS@GSH NPs of 2.7 nm were obtained after reflux for 3 h, and CdS@GSH NPs of 3.7 nm were obtained after reflux for 10 h.

The calculation of the size of CdS@GSH NPs was based on the following equation according to Yu et al. (2003):

$$\mathrm{D=}\left(\mathrm{-6}\mathrm{.6521\times 1}0^{\mathrm{-8}}\right)\lambda^{3}+\left(1\mathrm{.9557\times 1}0^{\mathrm{-4}}\right)\lambda^{2}-\left(9\mathrm{.2352\times 1}0^{\mathrm{-2}}\right)\mathrm{\lambda +}\left(\mathrm{13}\mathrm{.29}\right).$$ (1)

Here, *D* (nm) is the size of a given nanocrystal sample and *λ* (nm) is the wavelength of the first excitonic absorption peak of the corresponding sample. For CdS@GSH NPs, the first excitonic absorption peak is 377 and 411 nm.

PbS@GSH. PbS@GSH was prepared by the same procedure used for the preparation of CdS@GSH, except that Pb(ClO_4_)_2_·6H_2_O (0.88 g, 0.019 M) was used instead of Cd(ClO_4_)_2_·6H_2_O. The solution turned from colorless to black. PbS@GSH of different sizes can be obtained by controlling the heating time at 50°C. PbS@GSH NPs of 2.7 nm were obtained after reflux for 1.5 h, and PbS@GSH NPs of 3.7 nm were obtained after reflux for 7.5 h.

The calculation of the size of PbS@GSH NPs was based on the following equation which was obtained by fitting the experimental data during the synthesis process (Supplementary Figure S9):

λ=683.53+83.03D,2.35 nm≤D≤4.21 nm. (2)

Here, *D* (nm) is the size of a given nanocrystal sample and *λ* (nm) is the wavelength of the first excitonic absorption peak of the corresponding sample. For PbS@GSH NPs, the first excitonic absorption peak is 908 and 990 nm, which is inconsistent with what was reported by Cheng and Li (2017) (the first excitonic absorption peak is 852 nm for 2.7 nm PbS@GSH NPs and 1085.8 nm for 3.7 nm PbS@GSH NPs according to the equation in the reference), but the linear trend is consistent. The difference in peak wavelengths is mainly due to the difference in the background materials, where the large difference in the background dielectric coefficient causes different polarization effects on the surface of the quantum dots, which has an impact on the peak wavelengths (in the study by Cheng and Li (2017), PbS NPs were dispersed in toluene; but in this paper, PbS NPs were dispersed in the water phase).

Purification of CdS@GSH and PbS@GSH NPs. Isopropanol (IPA) and as˗made CdS@GSH and PbS@GSH suspensions (i.e., hardcore size of 2.7 and 3.7 nm) were mixed at a volume ratio of 1:1 (IPA:NP) and centrifuged at 7,000 rpm for 5 min. After rapid removal of the supernatant, the viscous transparent NP drops were directly obtained. Residual IPA was removed in a vacuum desiccator for 24 h at room temperature.

### Preparation of CdS@GSH and PbS@GSH Hydrogels

CdS@GSH and PbS@GSH hydrogels were obtained by adding 24 w/w% ultra-pure water into the dried samples. All performance tests were carried out after a maturation time of 8 h. All hydro-NPs were frozen in liquid nitrogen for 30 min and subsequently transferred to a cooling condenser at -80°C until all samples were completely dry.

### Rheological Measurements

The rheometer (TA DHR˗2) is equipped with parallel fixed plates (diameter 25 mm) for use at 25°C with a gap of 1 mm. The gels were attached directly to the plate. The top plate was carefully lowered in order to protect the hydrogel structure. The bottom plate was allowed to stand until the force practically dissipated to zero. The samples were covered with silicon oil to prevent water evaporation during the testing process. The frequency dependence of the oscillatory shear deformation of the NP hydrogel was characterized at a fixed strain of 0.05%. The viscoelasticity and shear dynamics of NP gels were measured and characterized with an oscillatory strain sweep, which was carefully performed from low to high strain in the range of 0.01–25% at a frequency of 1 Hz or 6.28 rad/s with the NP hydrogel loaded between the plates.

### NMR Analysis

CdS@GSH and PbS@GSH NPs (∼50 mg) and Cd/Pb-GSH compounds (∼25 mg) were dispersed in 500 μL deuteroxide (99% D atom) and then transferred into a Wilmad NMR tube. Solution-state NMR experiments including ^1^H spectrum, ^13^C spectrum, ^1^H-^13^C HSQC (heteronuclear single quantum coherence), 2D COSY [(homonuclear chemical shift) correlation spectroscopy], 2D NOESY (nuclear Overhauser effect spectroscopy), 2D ROESY (rotating-frame nuclear Overhauser effect correlation spectroscopy), and DOSY (diffusion-ordered spectroscopy) were carried on a Bruker Advance DRX 500 spectrometer with the ^1^H frequency at 500 MHz and controlled temperature at 298 K. All the ^1^H and ^13^C signals were distinguished by chemical shifts (*δ*) and integral area. The CdS@GSH and PbS@GSH NOESY measurements were done with a mixing time of 100 ms, and the mixing time of Cd/Pb–L-GSH complexes was 900 ms. The DOSY measurements were tested with the ledbpgp2s pulse sequence. A gradient pulse duration of 4.0 ms and a diffusion delay of 27 ms were used.

## Data Availability

The original contributions presented in the study are included in the article/Supplementary Material, further inquiries can be directed to the corresponding author.
