# Supplementary material for: Viscoelasticity Investigation of Semiconductor NP (CdS and PbS) Controlled Biomimetic Nanoparticle Hydrogels
Source: Front Chem. 2022 Jan 19;9:816944. doi: 10.3389/fchem.2021.816944 (PMC8807550; doi:10.3389/fchem.2021.816944)
Supplement: Supplementary file 1 [file DataSheet1.docx]

Supplementary Material

Viscoelasticity investigation of semiconductor NPs (CdS and PbS) controlled biomimetic nanoparticle hydrogels

**Dan Zhao^1*^, Wang Zhang^1^ and Zhi-Zhou Chen^2*^**

*^1^ School of Marine Sciences, Ningbo University, Ningbo, 315832, China*

*^2^College of Electrical and Electronic Engineering, Wenzhou University, Wenzhou 325035, China*

**Table** **S1.** Carbon and hydrogen content of Cd-GSH and Pb-GSH complexes

| Sample | Weight (mg) | C % | H % | Organic % | Inorganic % |
| --- | --- | --- | --- | --- | --- |
| Cd-GSH | 1.9818 | 19.7 | 3.45 | 45.32 | 54.68 |
| Pb-GSH | 2.0012 | 18.07 | 3.28 | 51.24 | 48.76 |
| CdS@GSH-2.7 | 1.9921 | 15.29 | 2.76 | 32.62 | 67.38 |
| CdS@GSH-3.7 | 2.0011 | 6.54 | 1.72 | 25.56 | 74.45 |
| PbS@GSH-2.7 | 1.9875 | 11.61 | 2.15 | 29.71 | 70.29 |
| PbS@GSH-3.7 | 1.9945 | 6.59 | 1.32 | 26.55 | 73.45 |


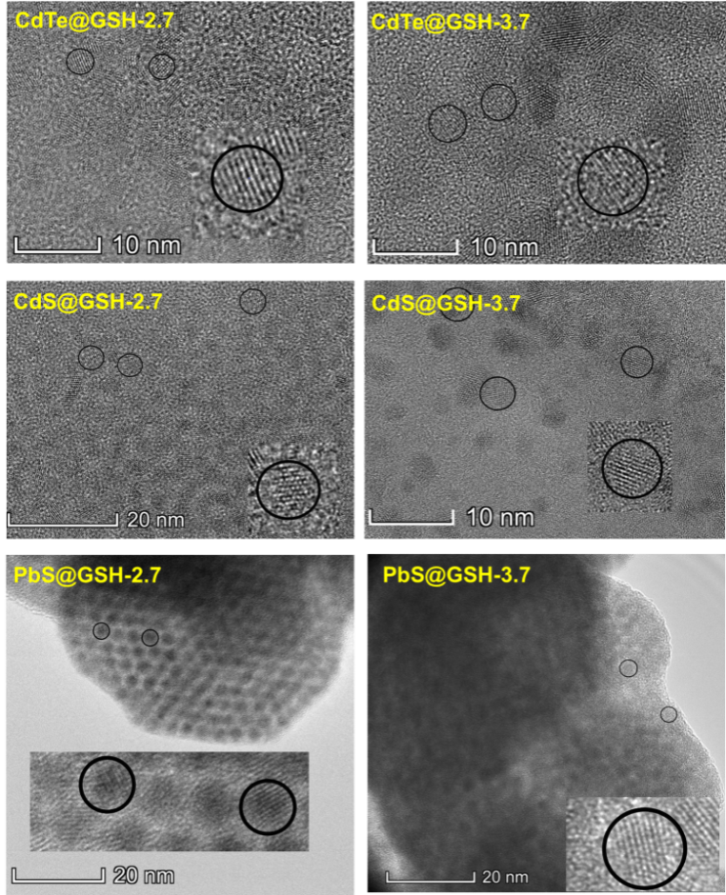


**Figure S1.** HRTEM images of 2.7 nm and 3.7 nm CdS@GSHandCdS@GSH NPs


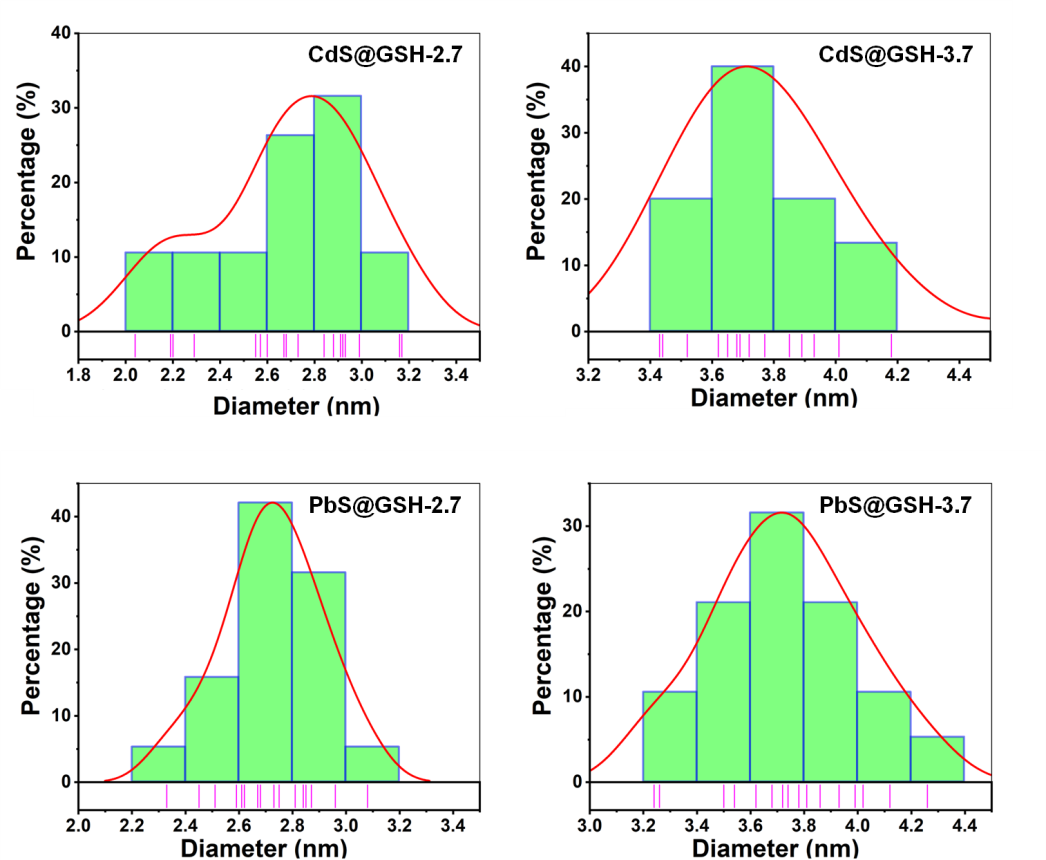


**Figure S2.** Size distributions of 2.7 and 3.7 nm CdS@GSH and PbS@GSH NPs.

**Figure S3.** PXRD patterns of CdS@GSH and CdS@GSH NPs at different size and pH value.

**
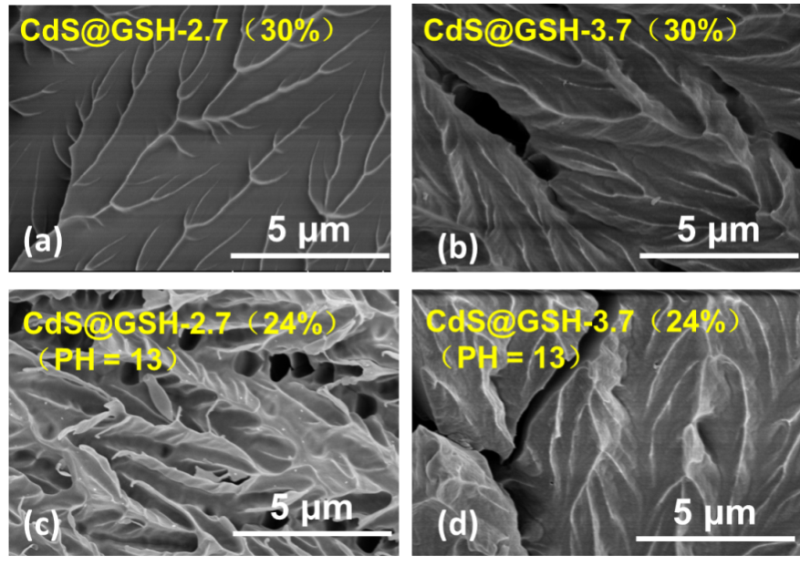
**

**Figure S4.** SEM images of CdS@GSH hydrogels under different concentration and pH value


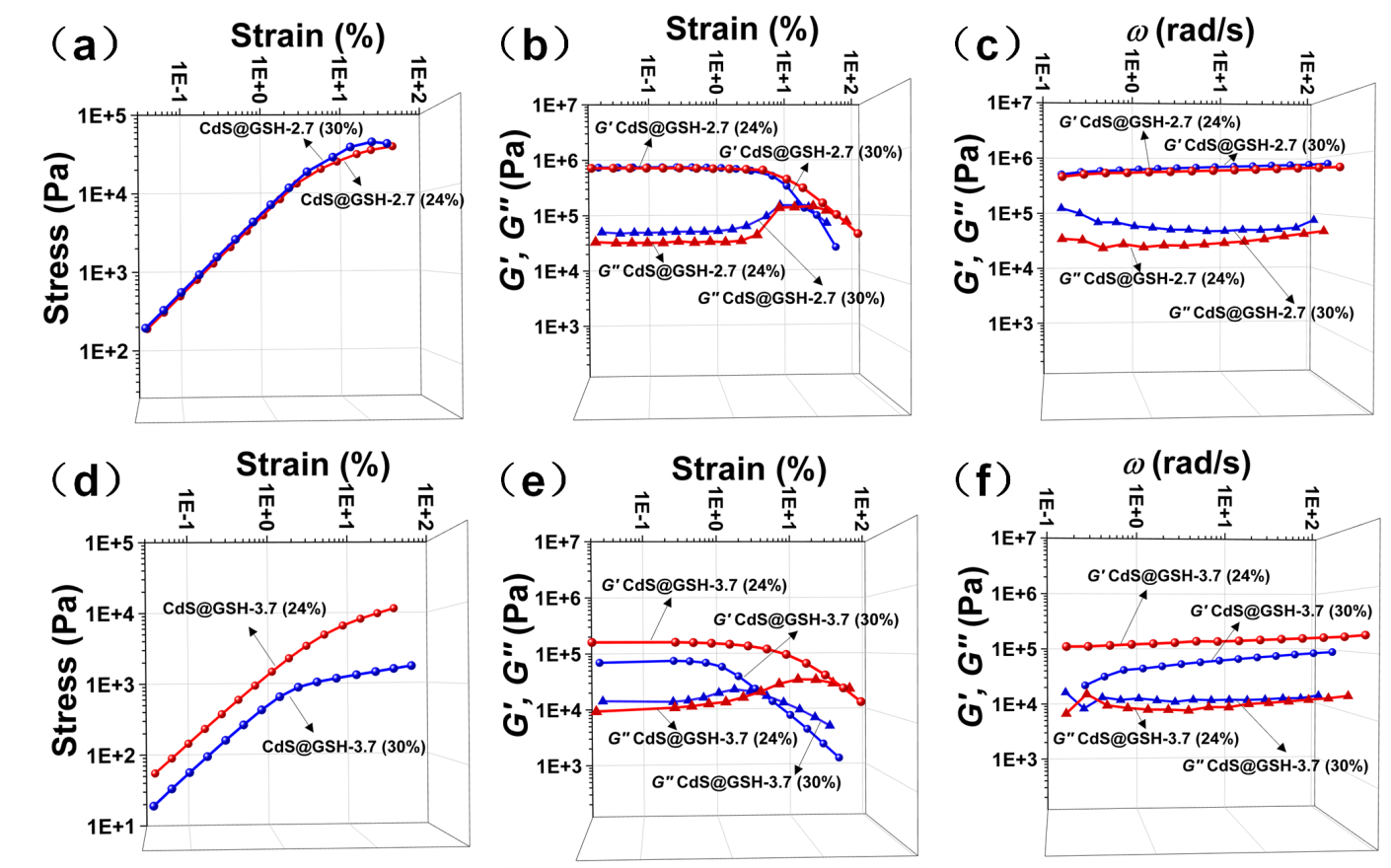


**Figure S5.** Tests of mechanical properties of CdS@GSH hydrogel at different concentration. Measurements of stress/strain ((a) and (d)), continuous step moduli/strain ((b) and (e)), and pheological dynamic oscillatory frequency sweep tests ((c) and (f)). Each measurement was performed at least three times from the same sample. The shear dynamics for each hydrogel was measured from low to high strain starting at 0.01–25% at a frequency of 6.28 rad/s. The rheological dynamic oscillatory frequency sweep measurements were performed with a parallel fixed plate (diameter 25 mm) at a strain value of 0.01%.


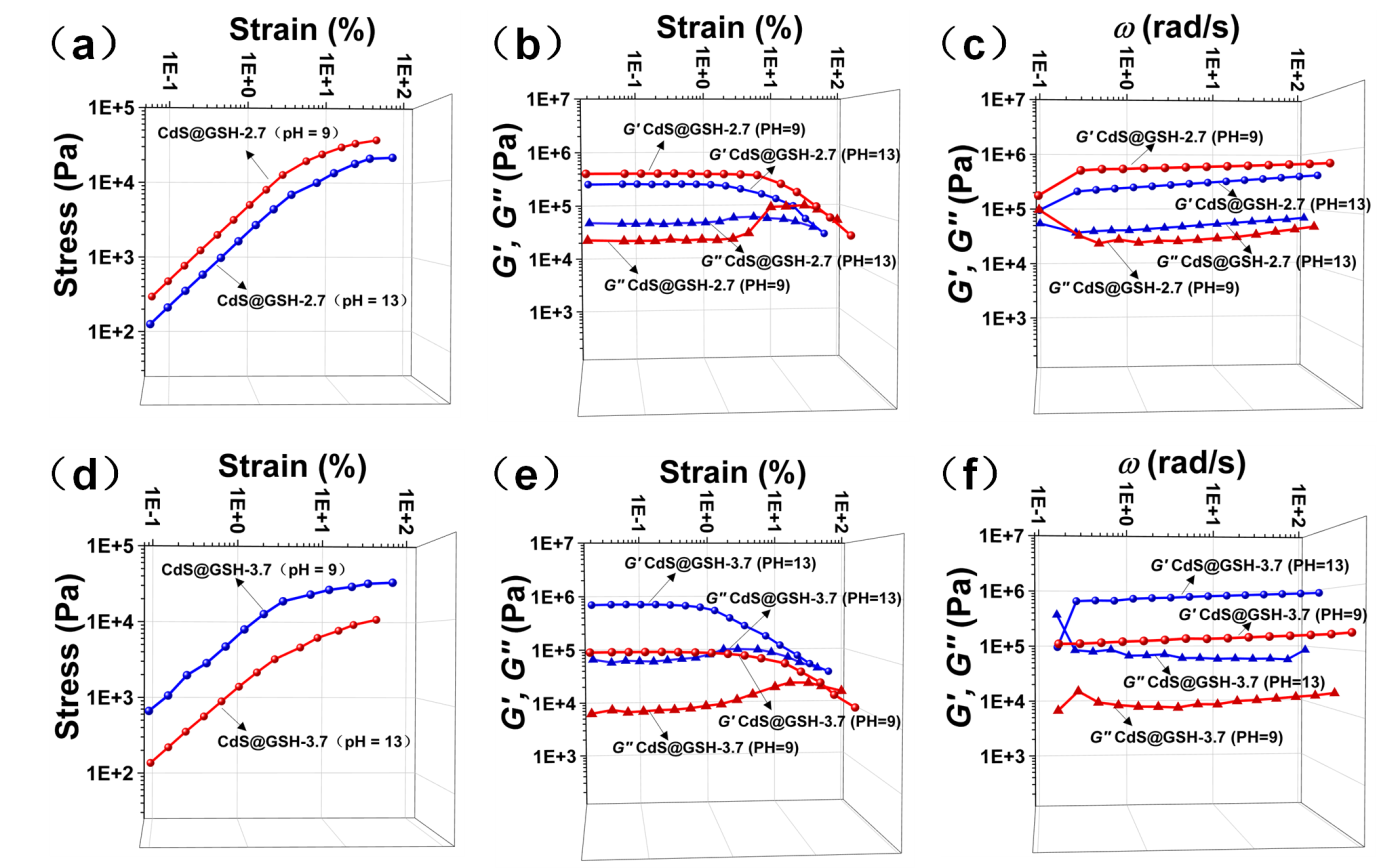


**Figure S6.** Tests of mechanical properties of CdS@GSH hydrogel at different pH value. Measurements of stress/strain ((a) and (d)), continuous step moduli/strain ((b) and (e)), and pheological dynamic oscillatory frequency sweep tests ((c) and (f)). Each measurement was performed at least three times from the same sample. The shear dynamics for each hydrogel was measured from low to high strain starting at 0.01–25% at a frequency of 6.28 rad/s. The rheological dynamic oscillatory frequency sweep measurements were performed with a parallel fixed plate (diameter 25 mm) at a strain value of 0.01%.

**Figure S7.** IR spectra of GSH,CdS@GSH and CdS@GSH NPs.


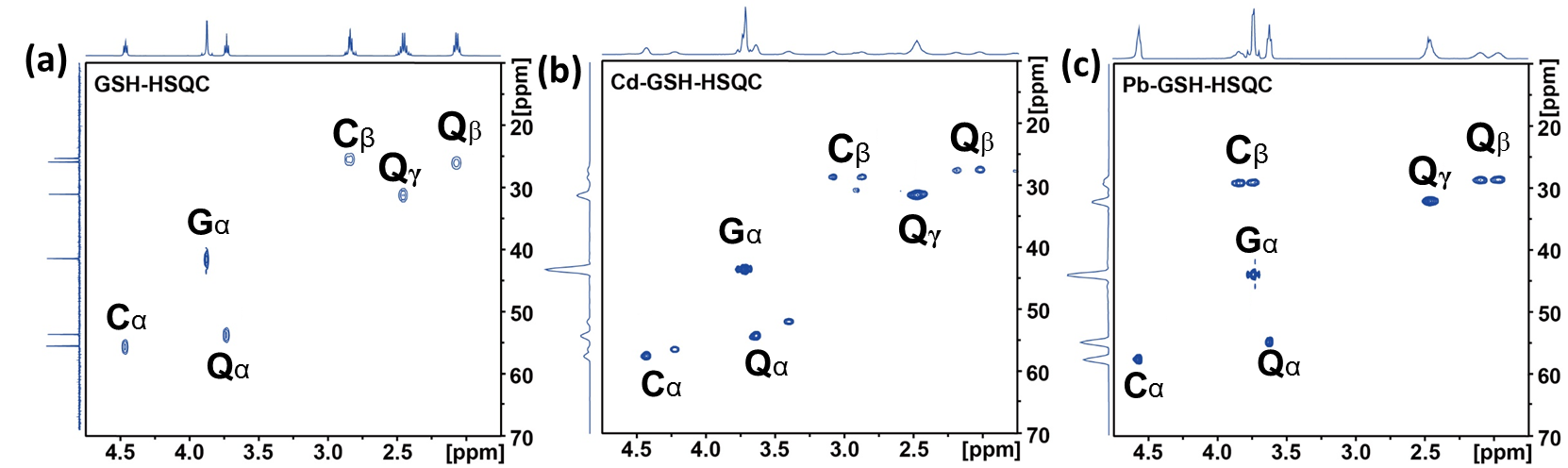


**Figure S8.** ^1^H˗^13^C HSQC spectra of (a) GSH, (b) CdS-GSH mixture and (c) PbS-GSH mixture.


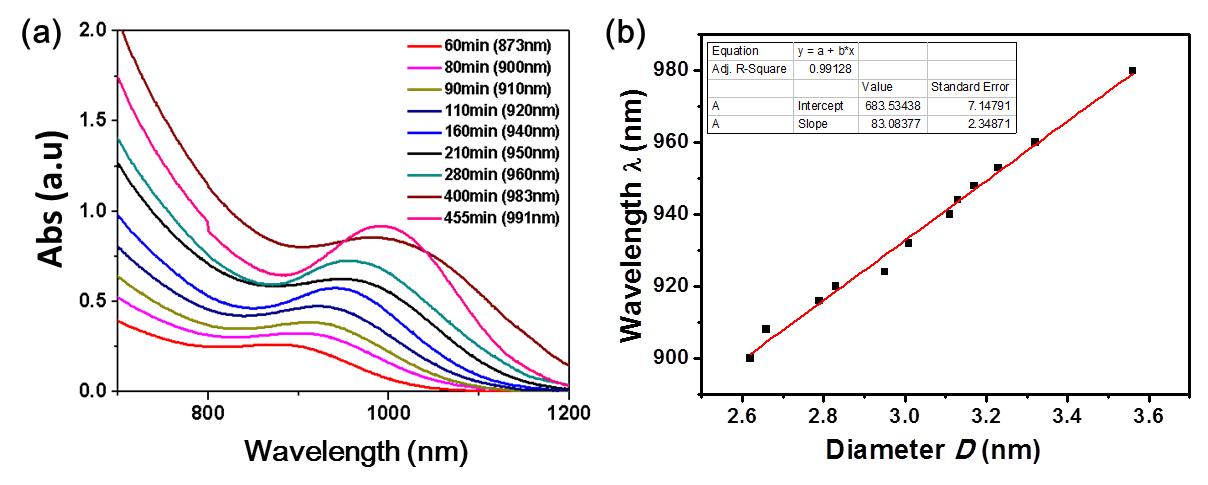


**Figure S9.** (a) Variation of absorption spectra of PbS@GSH NPs dispersions with the time, (b) Variation of the first absorption peak wavelength with PbS@GSH NPs diameter.
